# Erratum: Zahn et al. Manipulation of Medicinal Products for Oral Administration to Paediatric Patients at a German University Hospital: An Observational Study. *Pharmaceutics* 2020, *12*, 583

**DOI:** 10.3390/pharmaceutics13070939

**Published:** 2021-06-24

**Authors:** Julia Zahn, André Hoerning, Regina Trollmann, Wolfgang Rascher, Antje Neubert

**Affiliations:** Department of Paediatrics and Adolescent Medicine, Universitätsklinikum Erlangen, Friedrich-Alexander-Universität Erlangen-Nürnberg (FAU), 91054 Erlangen, Germany; andre.hoerning@uk-erlangen.de (A.H.); regina.trollmann@uk-erlangen.de (R.T.); wolfgang.rascher@uk-erlangen.de (W.R.)

The authors wish to make the following corrections to the affiliation and acknowledgments part [1]. The affiliation of all of the authors of “Department of Paediatrics and Adolescent Medicine, Universitätsklinikum Erlangen, 91054 Erlangen, Germany”, should be “Department of Paediatrics and Adolescent Medicine, Universitätsklinikum Erlangen, Friedrich-Alexander-Universität Erlangen-Nürnberg (FAU), 91054 Erlangen, Germany”. The updated “Acknowledgments” section is as follows “The authors would like to thank the nursing staff for a valuable exchange on the subject of manipulation and the opportunity to observe dispensing of medicines in clinical practice. The present work was performed in (partial) fulfilment of the requirements for obtaining the degree Dr. rer. biol. hum.”.

The authors would like to apologize for any inconvenience caused to the readers by these changes.
