# Supplementary material for: Manipulation of Medicinal Products for Oral Administration to Paediatric Patients at a German University Hospital: An Observational Study
Source: Pharmaceutics. 2020 Jun 23;12(6):583. doi: 10.3390/pharmaceutics12060583 (PMC7355957; doi:10.3390/pharmaceutics12060583)
Supplement: Supplementary file 1 [file pharmaceutics-12-00583-s001.pdf]

# Supplementary Materials: Manipulation of Medicinal Products for Oral Administration to Paediatric Patients at a German University Hospital: An Observational Study

**Julia Zahn *, André Hoerning, Regina Trollmann, Wolfgang Rascher and Antje Neubert ***

**Table S1**. Examples of active substances manipulated in this study categorised according to licensing status and preventability.

| License[1] | Preventability[2] | Active substance | n | Alternatives[3] |
|---|---|---|---|---|
| 1 – on-label | 1a – preventable | Amlodipine | 1 | Amlodigamma 5 mg tablets |
| | | Baclofen | 1 | Lioresal® 5 mg tablets |
| | | Cholecalciferol | 41 | Vigantol oil 20.000 IU/mL oral drops. |
| | | Hydrocortisone | 2 | Alkindi® 5 mg granules in capsules |
| | | Lamotrigine | 3 | Lamictal 100 and 25 tablets |
| | | Levothyroxine | 4 | Eferox oral solution 100 µg/5 mL |
| | | Prednisolone | 6 | Okrido 6 mg/mL oral solution, Decortin H 10 mg tablets |
| | | Ursodeoxycholic acid | 2 | Ursofalk® 250 mg/5 mL suspension |
| | 1a – non-preventable | Acetylsalicylic acid | 2 | - |
| | | Ferrous gluconate | 5 | - |
| | | Lamotrigine | 2 | - |
| | | Metoprolol succinate | 1 | - |
| | | Omeprazole | 11 | - |
| | | Oxcarbazepine | 4 | - |
| | | Valproic acid | 4 | - |
| 2 – off-label | 2a – preventable | Brivaracetam | 2 | Briviact® 25mg/50mg tablets |
| | | Clonazepam | 2 | Rivotril® 2,5 mg/mL oral drops |
| | | Dexamethasone | 4 | Infectodexakrupp® 2 mg/5 mL liquid |

| | | | |
|---|---|---|---|
| | Gabapentin | 2 | Gabaliquid® 50mg/mL, Neurontin 100mg hard capsules |
| | Hydrocortisone | 1 | Alkindi® 5 mg granules in capsules |
| | Melatonin | 1 | Slenyto® prolonged-release tablets |
| 2b – non-preventable | Azathioprine | 2 | - |
| | Baclofen | 5 | - |
| | Clonidine | 5 | - |
| | Enalapril | 2 | - |
| | Esomeprazole | 2 | - |
| | Potassium bromide | 4 | - |
| | Omeprazole | 13 | - |
| | Phenobarbital | 11 | - |
| | Topiramate | 10 | - |
| | Vigabatrin | 6 | Kigabeq® soluble tablets (available only from 01 July 2019) |

[1] License of the manipulation according to SmPC; [2] Preventability by using an alternative, commercially available medicinal product; [3] Alternative commercially available medicinal product(s) that would have prevented the manipulation.
